# Prevalence of Tobacco Use in Urban, Semi Urban and Rural Areas in and around Chennai City, India

**DOI:** 10.1371/journal.pone.0076005

**Published:** 2013-10-01

**Authors:** Kolappan Chockalingam, Chandrasekaran Vedhachalam, Subramani Rangasamy, Gomathi Sekar, Srividya Adinarayanan, Soumya Swaminathan, Pradeep Aravindan Menon

**Affiliations:** National Institute for Research in Tuberculosis, Chennai, India; McGill University, Canada

## Abstract

**Background:**

Tobacco use leads to many health complications and is a risk factor for the occurrence of cardio vascular diseases, lung and oral cancers, chronic bronchitis etc. Almost 6 million people die from tobacco-related causes every year. This study was conducted to measure the prevalence of tobacco use in three different areas around Chennai city, south India.

**Methods:**

A survey of 7510 individuals aged > = 15 years was undertaken covering Chennai city (urban), Ambattur (semi-urban) and Sriperumbudur (rural) taluk. Details on tobacco use were collected using a questionnaire adapted from both Global Youth Tobacco Survey and Global Adults Tobacco Survey.

**Results:**

The overall prevalence of tobacco use was significantly higher in the rural (23.7%) compared to semi-urban (20.9%) and urban (19.4%) areas (P value <0.001) Tobacco smoking prevalence was 14.3%, 13.9% and 12.4% in rural, semi-urban and urban areas respectively. The corresponding values for smokeless tobacco use were 9.5%, 7.0% and 7.0% respectively. Logistic regression analysis showed that the odds of using tobacco (with smoke or smokeless forms) was significantly higher among males, older individuals, alcoholics, in rural areas and slum localities. Behavioural pattern analysis of current tobacco users led to three groups (1) those who were not reached by family or friends to advice on harmful effects (2) those who were well aware of harmful effects of tobacco and even want to quit and (3) those are exposed to second hand/passive smoking at home and outside.

**Conclusions:**

Tobacco use prevalence was significantly higher in rural areas, slum dwellers, males and older age groups in this region of south India. Women used mainly smokeless tobacco. Tobacco control programmes need to develop strategies to address the different subgroups among tobacco users. Public health facilities need to expand smoking cessation counseling services as well as provide pharmacotherapy where necessary.

## Introduction

Tobacco is used in forms either designed for smoking or for non-smoking routes. Tobacco use alone is currently ranked fourth in the world in its contribution to years of life lost [1]. The smokeless forms of tobacco are used mostly in developing countries, especially those in south East Asia. In India, tobacco is smoked as a cigarette, beedi (a thin, Indian cigarette filled with tobacco flake and wrapped in a tendu leaf tied with a string at one end) cheroot (a cigar cut square at both ends), or in a pipe. The smokeless forms of tobacco are chewed as raw tobacco leaves or panmasala (a mixture of arecanut, tobacco and other ingredients), or inhaled as snuff. Tobacco use is considered to be one of the chief preventable causes of death in the world. Globally, smoking causes about 71% of lung cancer, 42% of chronic respiratory diseases and about 10% of cardiovascular disease. It is responsible for 12% and 6% of male and female deaths respectively [2]. In India, it was reported in 1990 that 1.5% of the total deaths were tobacco related [3]. Tobacco continues to kill 6 million people each year globally, including more than 600000 non-smokers who die from exposure to tobacco smoke [4]. The chewing of tobacco products is a risk factor for oral cancers [5]. Further, tobacco consumption was found to be higher among the lower socio-economic groups [6].

Basic data on tobacco use at the national level is available from the National Family Health Surveys [7] (a large scale, multi-round survey conducted in a representative sample of households in India). To estimate tobacco use among school children in the 8^th^ grade to 10^th^ grade (ages 13 to 15 years) and adults (of age >15 years), nationwide Global Youth Tobacco Survey (GYTS) [8], [9] and Global Adult Tobacco Survey (GATS) have been conducted [10]. However, there is limited data on the social and behavioural factors that influence the use of various forms of tobacco and attitudes of tobacco users towards quitting the habit.

Accordingly a study was planned and conducted in urban, semi-urban and rural populations residing in and around Chennai city in the southern Indian state of Tamilnadu to estimate: a) the prevalence of use of various forms of tobacco (cigarette/beedi smoking, smokeless chewing tobacco or snuff) and study the factors that influence use of tobacco. Behavioural patterns of tobacco use among the adult population are described, based on their knowledge, attitude and practice as captured in the questionnaire adapted from GYTS and GATS.

## Materials and Methods

### Study population

This study was conducted in selected wards of Chennai city (urban), Ambattur municipality (semi-urban) and villages of Sriperumbudur taluk (Rural) among individuals aged > = 15 years.

### Study design

The national estimate of prevalence of consumption of tobacco in any form among 15–54 year old men was 57.6% and that among women was 10.8%, with an overall prevalence of 28.3% [7]. With a relative precision of 10%, coverage of 90% and design effect of 2, the required sample size was estimated at 2141. This sample size was used in each of the three study areas (urban, semi-urban, and rural).

Twenty percent of the sampling units (villages in rural area and wards in urban and semi urban areas) were randomly selected and the sample size was equally distributed among the total units in each centre. Within the selected unit, households were selected as follows: from among the subunits of the rural area (main village, colony and hamlets), one unit was randomly selected and within that unit the survey began from the household in the northwest corner and proceeded continuously till the required sample for that unit was completed. All adults aged 15 years and above within that household were included in the survey. For the urban and semi-urban areas, the subunits were the streets; from those subunits, one street each was randomly selected from slum and non slum localities and the survey commenced from the north west corner of the selected street.

The survey was carried out between September 2009 and September 2011.

### Data collection

Data on tobacco use was collected using a questionnaire adapted from both Global Youth Tobacco Survey and Global Adults Tobacco Survey questionnaires [11] to collect information on knowledge, attitude and perception of tobacco use among the participants. Data on tobacco use, the type of tobacco, duration of usage, age at which the tobacco use started etc., were collected using this questionnaire. Written informed consent was obtained from all participants.

### Ethics statement

The study was approved by the Institutional Ethics Committee (of National Institute for Research in Tuberculosis (No: NIRT IEC 200902)). In case the participant was in the age group of 15–18 years, written consent was obtained from his/her parents or guardian. The consent procedure was approved by the ethics committee.

### Data management and Statistical analysis

The data were scrutinised and computerized, using double data entry, edited and corrected for discrepancy and missing information. Data were analysed using SPSS/PC version 14.0.

Estimates of prevalence of all forms of tobacco (with smoke and/or smokeless) use along with their 95% confidence intervals were computed. For analysis, it was decided to combine those individuals who used both forms of tobacco along with the smoking group as it was earlier reported that they have the same profile as that of smokers [12]. Chi square test was used to look at the association between groups and to see the trend with age. P value <0.05 was considered significant.

#### Multivariate analysis

It is known that there is preference for any one form of tobacco, either smoke or smokeless. We wanted to explore the factors that influence this preference. For this multinomial logistic regression analysis was carried out with the type of tobacco user as the outcome variable. Accordingly, the outcome variable had 3 values namely 0 – for non users, 1-tobacco smokers and 2 for smokeless tobacco users. The predictor variables considered were, age, gender, locality (slum or non slum), area (urban, semi-urban or rural) and alcoholism.

#### Cluster Analysis

We attempted to study the patterns of tobacco use among adults based on their responses to the questions in the questionnaire. Accordingly they were grouped into different domains (as per GATS) like (a) Use of tobacco (b) User’s attitude towards stopping tobacco (c) Exposure to second hand smoking and (d) User’s knowledge on messages/media contents on tobacco use (Table 1). Cluster analysis was undertaken using SPSS v14.0 to study the patterns among current tobacco users with respect to variables (Table 1) under the above mentioned domains.

**Table 1:** Variables and domains they represent.

Variable names	Domains and the Questions in the questionnaire
**Domain 1 -Use of tobacco**	
A3	During the past 30 days (one month), on how many days did you try tobacco product
A4	During the past 30 days (one month), on the days you tried tobacco product, how many did you use on an average
**Domain 2-User’s attitude towards stopping tobacco**	
A5	Do you want to stop tobacco habit now
A6	Have you ever tried to stop tobacco habit
A7	How long ago did you stop tobacco use
A8	What was the main reason you decided to stop using tobacco
A9	Do you think you would be able to stop tobacco use if you wanted to
A10	Have you ever received help or advice to help you stop tobacco use
**Domain 3-Exposure to second hand smoking**	
B1	Do you think the tobacco smoking by other people is harmful to you
B2	During the past 7 days, on how many days have people your home, in your presence used tobacco smoking in
B3	During the past 7 days, on how many days have people used tobacco smoking in your presence, in places other than in your home
**Domain 4-User's knowledge on messages/media contents on tobacco use**	
B4	Are you in favor of banning all types of tobacco smoking in public places (such as in restaurants, in buses, streetcars, and trains, in schools, on playgrounds, in gyms and sports arenas, in discos)
B5	During the past 30 days (one month), how many anti-tobacco media messages (e.g., television, radio, billboards, posters, newspapers, magazines and movies) have you seen

Hierarchical cluster analysis was carried out to get an idea about the naturally occurring clusters in the data in terms of the variables. Ward’s technique was used as it provides the cluster centres, a measure to explore the optimal number of clusters. Then, with this information, K-means cluster analysis was performed, based on cluster centres from the hierarchical analysis, to refine the solution further. While using K means cluster we have used list wise exclusion option, which used only those cases that had responses for the clustering variables (Table 1) used in the analysis. Also, only current tobacco users were included in the analysis.

## Results

The total sample size from all the three areas was 7,510 (Table 2). Fifty three percent were females, and the 15–44 year age group was predominant in all three areas (Rural (R) 68%, Semi – Urban (SU) 68%, Urban (U) 59%).

**Table 2:** Age-Gender Distribution of the study population.

Age groups(in years)	Rural		Semi – Urban		Urban		Combined		All
	F	M	F	M	F	M	F	M	
15 – 17	79	64	52	65	52	59	183	188	371
18 – 24	250	220	171	185	160	165	581	570	1151
25 – 34	342	291	307	263	314	256	963	810	1773
35 – 44	283	250	258	241	307	261	848	752	1600
45 – 54	215	169	161	169	263	213	639	551	1190
55 – 64	143	111	134	103	172	167	449	381	830
≥ 65	94	97	66	79	129	130	289	306	595
Total	1406	1202	1149	1105	1397	1251	3952	3558	7510

F – females; M-males.

### Overall tobacco use

Combining all the three areas, the overall prevalence of tobacco use was 21.4%, this being 23.7%, 20.9% & 19.4% respectively in rural, semi-urban and urban areas; the differences were statistically significant (p value < 0.001) (Table 3). Tobacco smoking prevalence was 13.5% (R-14.3%, SU-13.9%, U-12.4%). The corresponding prevalence for smokeless tobacco use was 7.8% (R-9.5%, SU-7.0%, U-7.0%).

**Table 3:** Prevalence of tobacco use in the study population.

Basic characteristics	Prevalence of tobacco use (%)			Total surveyed (N)	P value*
	Tobacco smoking	Smokeless tobacco	Any form of tobacco		
	n (% of N)	n (% of N)	n (% of N) (95% CI)		
**Age group**					
15–34	279 (8.5)	178 (5.4)	457 (13.9)	3295	<0.001
			(12.7–15.0)		
35–54	488 (17.5)	234 (8.4)	722 (25.9)	2790	
			(24.3–27.5)		
55 & Above	248 (17.4)	177 (12.4)	425 (29.8)	1425	
			(27.4 – 32.2)		
**Sex**					
Female	4 (0.1)	193 (4.9)	197 (5.0)	3952	<0.001
			(4.3 – 5.7)		
Male	1011 (28.4)	396 (11.1)	1407 (39.6)	3558	
			(38.0 – 41.2)		
**Area**					
Urban	328 (12.4)	185 (7.0)	513 (19.4)	2648	<0.001
			(17.9 – 20.9)		
Semi Urban	314 (13.9)	158 (7.0)	472 (20.9)	2254	
			(19.3 – 22.6)		
Rural	373 (14.3)	246 (9.5)	619 (23.7)	2608	
			(22.1 – 25.4)		
**Locality**					
Non Slum	610 (12.0)	321 (6.3)	931 (18.3)	5095	<0.001
			(17.2 – 19.3)		
Slum	405 (16.8)	268 (11.1)	673 (27.9)	2416	
			(26.1 –29.6)		
Total	1015 (13.5)	589 (7.8)	1604 (21.4)	7510	
			(20.4 – 22.3)		

* Significance between variables (example; Age, Sex, Area and Locality) vs. any tobacco use

**Figure 1:**
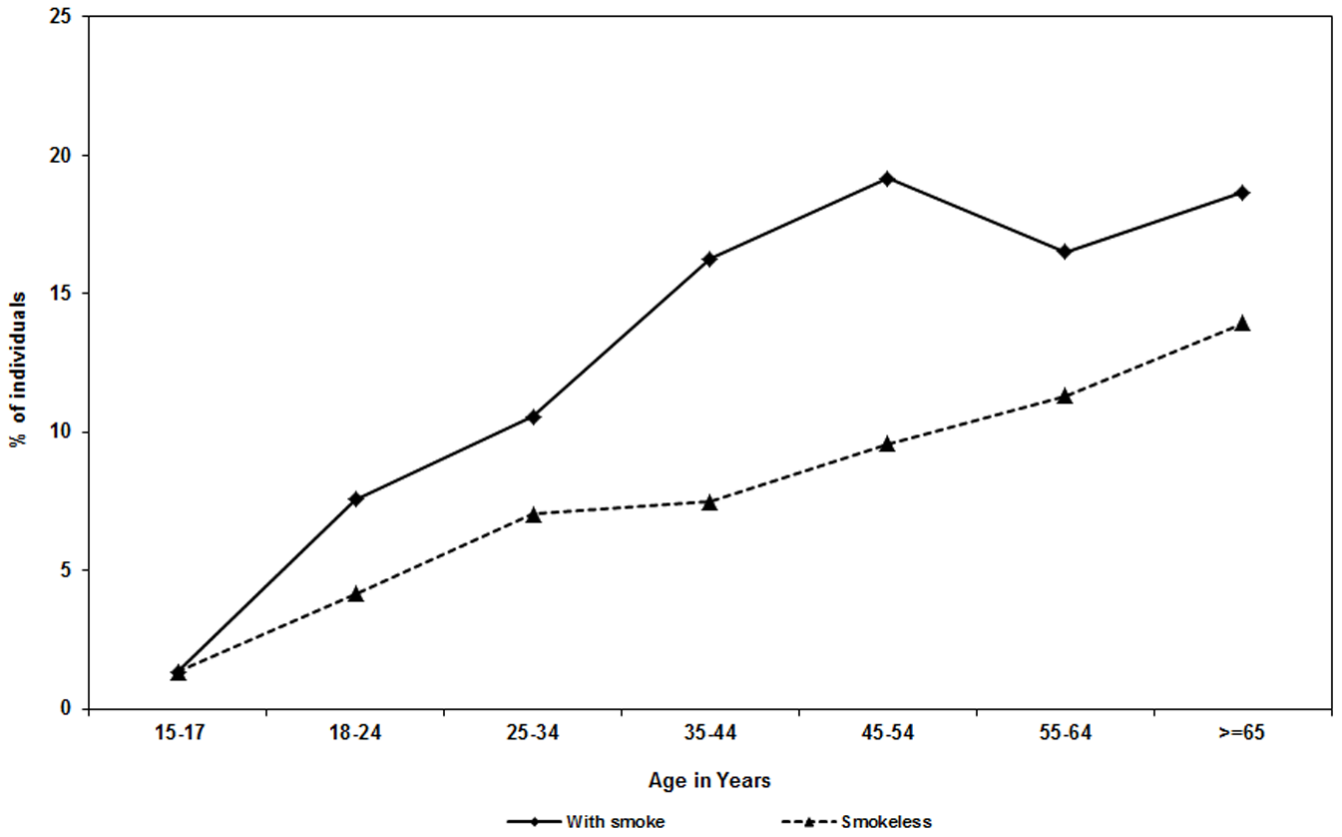
Prevalence of tobacco use by age.

The prevalence of tobacco increased with age (Figure 1) and this increase was found to be statistically significant (Chi-square for trend = 189, P value <0.001). Prevalence of tobacco use was significantly higher (39.6%) among males when compared to females (5.0%) (P value <0.001). A significantly higher prevalence of tobacco use was observed in slum areas (27.9%) when compared to that in non-slum areas (18.3%) (P value <0.001). A significantly higher proportion of males resorted to tobacco smoking (28.4%) and smokeless tobacco (11.1%) when compared to the same (0.1%) and (4.9%) respectively among females (P value <0.001).

### Among tobacco smokers

Seventy seven percent of smokers used cigarettes and 34% used beedis (Table 4). Sixty percent started smoking between the age of 15 to 24 years, and 44% had been smoking for more than 20 years. Sixty nine percent smoked daily while 52% smoked less than five times per day. Sixty eight percent wanted to stop smoking, and 27% had stopped smoking for a duration of less than one year. The main reason provided for stopping smoking habit was intention to improve health.

**Table 4:** Responses to queries put across to male tobacco smokers only (n = 1011).

Queries	Tobacco smoking (%) #			
	
What is the Type of tobacco used? *	Cigarette	Beedi	Pipe	Cigar
	77	34	0	0
Age at which started	5–14y	15–24y	25–34y	35y and above
	7	60	25	9
Duration of Tobacco use	≤ 5y	6–10y	11–20y	20+ y
	20	16	20	44
How many days Tobacco used in the last 30 days?	Not used	1–10 days	11–29 days	All 30 days
	17	9	5	69
In the last 30 days on an average how many items used/day?	< 5/day	6 –10/day	11–20/day	>20/day
	52	23	13	12
Do you want to stop the habit now?	Yes		No	
	68		32	
Have you ever tried to stop the habit?	Yes		No	
	45		55	
Duration of stopping (if any) the habit?	Not stopped	< 1year	1–3 years	> 3 Years
	68	27	4	2
Reason for stopping the habit*	To improve health	Family	Friends	Others
	73	14	7	11

#: M-Males (n = 1011); F-Females (n = 04); females excluded because of very few numbers.

* -Multiple answers.

### Among smokeless tobacco users

Chewing of raw tobacco leaves (57%) was the most common type of smokeless tobacco use, followed by the chewing of panmasala (29%) (Table 5). Forty percent had been using smokeless tobacco products for ≤5 years, with 73% using one or other smokeless tobacco product on all days in a month. Seventy eight percent of the users used one or other product ≤ 5 times per day. Fifty four percent wanted to stop the habit, however, only 33% of the users ever tried to stop the habit during the entire course of their smokeless tobacco use. Among the smokeless tobacco users, 23% had stopped the habit for less than 1 year. The main reason given for stopping the habit was to improve the health.

**Table 5:** Responses to queries put to smokeless tobacco users only (n = 589).

Queries#	Smokeless Tobacco user (%)											
	
	M	F	T	M	F	T	M	F	T	M	F	T
What is the Type of Tobacco used? *	Chewing			Snuff			Panmasala			Others		
	49	71	57	11	28	17	42	2	29	3	1	2
Age at which started	5–14y			15–24y			25–34y			35y and above		
	4	5	4	43	27	38	28	22	26	25	45	31
Duration of Tobacco use	≤ 5y			6–10y			11–20y			20+ y		
	47	27	40	21	16	19	14	11	13	19	46	28
How many days Tobacco used in the last 30 days?	Not used			1–10 days			11–29 days			All 30 days		
	9	9	9	9	9	9	11	4	9	70	78	73
In the last 30 days on an average how many items used/day?	< 5/day			6 –10/day			11–20/day			>20/day		
	77	79	78	17	14	16	4	3	4	2	4	3
Do you want to stop the habit now?	Yes						No					
	M-58		F-45		T-54		M- 42		F- 55		T-46	
Have you ever tried to stop the habit?	Yes						No					
	M-37		F-22		T-33		M-62		F-78		T-67	
Duration of stopping (if any) the habit?	Not stopped			< 1year			1–3 years			> 3 Years		
	69	85	74	28	14	23	2	1	2	1	0	1
Reason for stopping the habit*	To improve health			Family			Friends			Others		
	70	73	71	18	19	18	4	4	4	14	19	15

#:M- Males (n =  396); F-Females (n = 193); T-Total (n = 589);

* - Multiple answers.

### Awareness on second hand/passive smoking

Of the survey participants, 93% felt that smoking by others was harmful to them (Table 6). However, only 20% said that someone had smoked in their presence in their home within the last 7 days. But 51% said that someone had smoked in their presence outside their home during the same period. Ninety nine percent were in favour of banning all types of smoking in public places. Ninety two percent had seen anti-tobacco messages in the media during the past one month.

**Table 6:** Responses to queries put to all participants (n = 7510).

Queries	Responses%		
Is tobacco smoking by others harmful to you?	Yes	No	No idea
	93	3	4
During the past 7 days, on how many days have people used tobacco smoking in your home, in your presence?	0 days	1–4days	5–7days
	80	9	11
During the past 7 days, on how many days have people used tobacco smoking in places other than your home, in your presence?	0 days	1–4days	5–7days
	49	31	20
Are you in favor of banning all types of tobacco smoking in public places?	Yes	No	No idea
	99	1	0
During the past 30 days how many anti-tobacco media messages have you seen?	A lot	Sometimes	Never
	42	50	8

### Knowledge and attitude among adolescents

An additional 17 questions were asked to persons aged 15 to 18 years (n = 482). Two thirds said that their parents did not smoke. Ninety three percent answered that they would not accept any tobacco item offered to them even by their best friends. Forty two percent said that they had discussed the harmful effects of tobacco smoking in their homes, and 96% said that they would not start smoking in the next 5 years. Sixty nine percent felt that it would be difficult to quit smoking once started. Forty two percent replied that smokers would be uncomfortable in parties and celebrations, 37% felt that boys who smoked tobacco were less attractive whereas 49% felt that girls who smoked were less attractive. Ninety three percent replied that tobacco use was harmful to health.

### Factors influencing tobacco use

Multinomial logistic regression analysis was done taking tobacco user as the dependent variable. Here non users were considered as the reference group. Age, gender, locality, area and alcoholism were found to be significant. The fitted regression model was significant (Chi square =  3121; P value = 0.000) and the model could correctly classify 85% of the observations, suggesting a good fit of the model.

As observed earlier, it was seen for both groups of users, that there was a significantly higher risk of tobacco use among those who lived in slum localities, rural areas, among males, older persons and those who consumed alcohol. The adjusted odds ratios for those who smoked and those used only smokeless tobacco are shown in Table 7. It may be seen that the odds of using tobacco (with smoke or smokeless) was significantly lower in urban areas when compared to rural areas.

**Table 7:** Factors associated with the use of tobacco.

Variables	Among tobacco smokers			Among smokeless tobacco users		
	Adj. odds	Z value	P value	Adj. odds	Z value	P value
	(95% CIs)			(95% CIs)		
**Locality**						
Non slum	1^a^			1^a^		
Slum	1.91	6.97	0.00*	2.3	8.92	0.00*
	(1.6–2.3)			(1.9–2.8)		
**Area**						
Rural	1^a^			1^a^		
Semi urban	1.04	0.39	0.69	0.8	–2.33	0.02*
	(0.8 – 1.3)			(0.6–1.0)		
Urban	0.7	–3.01	0.00*	0.6	–4.14	0.00*
	(0.6–0.9)			(0.5–0.8)		
**Age**						
15–17	1^a^			1^a^		
18–44	5.8	3.77	0.00*	4.3	4.18	0.001
	(2.3–14.5)			(1.7–10.6)		
>44	18.6	6.25	0.00*	12.6	5.5	0.00*
	(7.5–46.2)			(5.1–30.9)		
**Gender**						
Female	1^a^			1^a^		
Male	192.9	10.43	0.00*	2.2	6.86	0.00*
	(71.7–518.7)			(1.7–2.7)		
**Alcohol use**						
No	1^a^			1^a^		
Yes	10.7	25.1	0.000	6.3	15.48	0.000
	(8.7–12.8)			(5.0–8.0)		

a: reference category.

* : significant at 5% level.

Similarly, the odds of tobacco use significantly increased with age, when all other variables are were held constant. A very high odds of 192.9 of smoking tobacco among males was due to the fact that women here hardly smoke however, when use of smokeless tobacco is concerned, the difference is not so large.

### Patterns of tobacco users

#### Among male Tobacco smokers

Of the 1011 male smokers, 839 were current smokers and among them only 567 individuals responded to all the relevant questions. Hence the analysis was restricted only to these 567 individuals. Cluster analysis showed that smokers broadly fell in 4 groups as shown in Table 8.

**Table 8:** Behavioural patterns among tobacco smokers based on their attitude, knowledge and practice.

Clusters	Group that consists of	In terms of questions (variables) in the questionnaire	No of cases in the clusters (%))
I	Smokers with none to advice or help to stop the habit of smoking	A3 and A10	200 (35.3)
II	Smokers well aware of harmful effects of tobacco and even support banning tobacco, smoke atleast 5 times a day	A4, B1, B4, B5	151 (26.6)
III	Smokers wanting to quit	A5, A6, A7, A9	44 (7.8)
IV	Smokers who are exposed to second hand smoking at home and outside	B2 and B3	172 (30.3)

Group one, were those smokers (35%) who did not receive any help or advice to quit the habit. The second group (27%) smoked in spite of knowing the harmful effects of tobacco. The third group (8%) wanted to quit the habit, but still continued to smoke. The fourth group (30%) consisted of those smokers exposed to passive or second hand smoking at home or outside.

#### Smokeless tobacco users

Of the 589 smokeless tobacco users only 534 were current tobacco users. Among these, only 393 had responses for all the clustering variables. Hence the analysis was restricted to these 393 individuals. Cluster analysis showed that the smokeless tobacco users formed 3 groups as shown in Table 9.

**Table 9:** Behavioural patterns among smokeless tobacco users based on their attitude, knowledge and practice.

Clusters	Group that consists of individuals who	In terms of questions (variables) in the questionnaire	No of cases in the clusters (%))
I	Daily use smokeless tobacco as there is none to advice or help to stop the habit	A3 an d A10	51 (13.0)
II	Though aware of harmful effects of tobacco, passive smoking, and support banning tobacco, use smokeless tobacco products atleast 5 times a day	A4, B1, B2, B3, B4, B5	124 (31.6)
III	Though want to quitting the habit, unable to do so currently but hopeful that they can stop anytime in future	A5 A6, A7, A9	218 (55.4)

Group one was those users (13%) who did not receive any help or advice to quit the habit. The second group (32%) knew the harmful effects of tobacco. The third group (55%) wants to quit the habit, but still continue to use smokeless tobacco.

The major difference observed between the patterns of smokers and smokeless tobacco users was that, the proportion that used tobacco unconditionally was smaller among smokeless tobacco users when compared to that among smokers. Further, a higher proportion of smokeless tobacco users wanted to quit and were aware of the ill effects of tobacco compared to the smokers.

## Discussion

In this survey conducted in and around Chennai city in South India, we observed that the overall prevalence of tobacco use (any form) was 21%, with a significantly higher prevalence in rural compared to semi urban and urban areas, similar to what has been observed in earlier studies in India [13], [14]. The proportion of individuals who used tobacco (any form) in Tamil Nadu as estimated in the latest NFHS-3 survey was estimated to be 40%, higher than our estimate particularly among males [15]. Higher tobacco use prevalence in rural areas was also observed in the national health survey. Further, the fact that the use of tobacco increased with age corroborates the findings of one of the earlier studies in India [16] and elsewhere [11]. The observation that tobacco use was associated with slum areas (i.e. with low standard of living conditions) also confirms earlier observations [14].

With respect to gender, our finding that tobacco usage was higher among males (39.6%) when compared to females (5.0%) also confirms the observations made in other studies on tobacco use [6], [9]–[11], [16] in India. However, the estimates of prevalence of tobacco use among males and females in our study are lower than those reported by these studies. While one study [16] reported tobacco use prevalence of 47% in men and 14% in women, another [14] reported a higher tobacco use prevalence of 50.2% for men and 15.5% for women. Interestingly, in an earlier survey conducted by NIRT in a rural area close to Chennai (Tiruvallur), tobacco smoking prevalence among males was reported to be 32% which is closer to our estimate [17].

The use of smokeless tobacco by women (though relatively small at 5% in our study) supports the finding that use of smokeless tobacco among women is prevalent in Asian counties like India, China and Bangladesh [11].

The findings of GATS was that majority of people in India smoked beedis and not cigarettes [11] which is different from our observation that the majority of men smoked cigarettes. This could be due to the fact that majority of smokers in this study were from the financially independent younger age groups. Our observation that majority of the tobacco users started using tobacco at the age of 15–24 years, also supports the fact the attainment of financial independence in this age group may lead to getting addicted to this habit.

With regard to quitting smoking, it was observed that 68% wanted to quit smoking and 45% even tried to stop smoking. Typically, [11] a very low proportion of smokers, (here only 2%) were successful in stopping the habit for more than 3 years. In the case of those using smokeless tobacco, 54% wanted to stop the habit currently, and 32% had already tried to stop the habit. However, majority of them continued to use tobacco and only 1% had stopped the habit for more than 3 years. This clearly underlines the addictive nature of tobacco use.

A unique feature of the current study is that we have attempted to use the information obtained on the knowledge, attitude and practice of current tobacco users to describe the behavioural patterns among smokers and smokeless tobacco users (Table 8). Based on these results, one could plan different intervention strategies to address the different needs of each group. Hence for tobacco smokers, the usage based intervention could be in these lines:

- Group 1 (cluster I) – represents one third of smokers (35%) who could not be reached by family or friends and therefore need to be targeted with better messaging and strengthen the intervention through peer group counseling, mass media messaging and whenever they come in contact with public health staff.
- Group 2 (Cluster II and III) – this one third of smokers (35%) are better informed of harmful effects of tobacco, even want to quit the habit but still they continue to smoke. With regard to intervention activities, this group will need intensive psychosocial and pharmacological strategies.
- Group 3 (Cluster IV)– this group of smokers (30%) consist of those who are exposed to passive smoking at home and outside and therefore need to be targeted through peer group education, both in the community and at the workplace.

Similarly for users of smokeless tobacco, based on their usage pattern (Table 9), the intervention activities may be as follows

- Group 1 (Cluster I): About 13% of the users who could not be reached, as mentioned earlier could be targeted through peer group counseling and mass media messaging.
- Group 2 (Cluster II and III) – A majority of users (87%) who were aware of the harmful effects of tobacco, need behavioural and enhanced psychosocial counseling strategies.

Another interesting observation was that young adults (aged 15–19 years) were more aware of the harmful effects of tobacco and it was reflected in the low prevalence in that age group (Figure 1). However, the prevalence increases with the age due to the addictive nature of the habit [3].

Addiction to tobacco use has to be viewed as a disease entity as it is found to be associated with respiratory and vascular diseases [18] and therefore addicted tobacco users need to be treated systematically through tobacco cessation clinics to wean away from the habit.

All these suggest that there is an urgent need to strengthen the existing public health anti-tobacco campaign, and tobacco cessation clinics are needed in all public health institutions to support the control of tobacco use. Our results on the behavioural patterns of tobacco user show that control strategies need to be group specific to make the tobacco cessation programme a success. Also, medical and para-medical graduates should be trained in tobacco cessation methods, so that the tobacco use habit can be effectively dealt with both in the private and public sectors. It was found in one of the studies that television is the single largest mass media reaching both genders and all age groups; hence a well-designed tobacco control campaign (similar to HIV awareness programs) will help curb tobacco use [19]. This finding is also supported by our study where, 92% of the participants had seen/heard anti tobacco messages in mass media instruments like newspaper, radio and television. These suggest that mass media may be an effective tool in our country in spreading anti-tobacco messages among the public. Further, though smoking in public places is prohibited, this law needs be implemented fully, with violators being punished as per the law. Such stringent measures will improve tobacco control in India.

## Conclusions

Tobacco use prevalence is higher among older age group, male population, and rural areas and in slums. Women mainly use smokeless tobacco. Since tobacco use is one of the preventable causes of morbidity and mortality, efforts should be made to control tobacco use in the country by improving the number and quality of services of tobacco cessation treatment, implementing usage based intervention strategies with counseling facilities and rigorous implementation of prohibition of smoking in public places.

## References

[1] Adejuwon GA (2009) Tobacco use and second hand smoke as risk factors for diseases in Nigeria: implications for collaborative research and multilevel tobacco control strategies. Afr J Med Med Sci 38 Suppl 221–29. 20229735

[2] WHO (1997) Tobacco or Health: A global status report. World Health Organization ISBN 92 4 156184 X (NLM classification: WM 290).

[3] Chaly PE (2007) Tobacco control in India. Indian J Dent Res 18: 2–5. 17347536 10.4103/0970-9290.30913

[4] WHO (2011) WHO report on global tobacco epidemic, 2011: Warning about dangers of tobacco. World Health Organization ISBN 978 92 4 156426 7 (NLM classification: WM 290).

[5] Critchley JA, Unal B (2003) Health effects associated with smokeless tobacco: a systematic review. Thorax 58: 435–443. 12728167 10.1136/thorax.58.5.435 PMC1746661

[6] Giovino GA, Henningfield JE, Tomar SL, Escobedo LG, Slade J (1995) Epidemiology of tobacco use and dependence. Epidemiol Rev 17: 48–65. 8521946 10.1093/oxfordjournals.epirev.a036185

[7] NFHS-2 (1998) India: National Family Health Survey (NFHS-2): Key findings. International Institute for Population Sciences, India: 1–14.

[8] WHO (2006) India Global Youth Tobacco Survey 2006.. Atlanta, United States: Centers for Disease Control and Prevention (CDC).

[9] Gajalakshmi V, Kanimozhi CV (2010) A survey of 24000 students aged 13–15 years in India: Global Youth Tobacco Survey 2006 and 2009. Tobacco Use Insights 3: 23–31.

[10] MOHFW (2010) Global Adult Tobacco Survey: Fact sheet: India: 2009-2010. International Institute for Population Sciences, Deonar, Mumbai, India.

[11] Giovino GA, Mirza SA, Samet JM, Gupta PC, Jarvis MJ, et al (2012) Tobacco use in 3 billion individuals from 16 countries: an analysis of nationally representative cross-sectional household surveys. Lancet 380: 668–679. 22901888 10.1016/S0140-6736(12)61085-X

[12] Gupta PC, Ray CS, Narake SS, Palipudi KM, Sinha DN, et al. Profile of dual tobacco users in India: an analysis from Global Adult Tobacco Survey, 2009-10. Indian J Cancer 49: 393–400. 10.4103/0019-509X.107746 23442404

[13] Neufeld KJ, Peters DH, Rani M, Bonu S, Brooner RK (2005) Regular use of alcohol and tobacco in India and its association with age, gender, and poverty. Drug Alcohol Depend 77: 283–291. 15734228 10.1016/j.drugalcdep.2004.08.022

[14] Subramanian SV, Nandy S, Kelly M, Gordon D, Davey Smith G (2004) Patterns and distribution of tobacco consumption in India: cross sectional multilevel evidence from the 1998-9 national family health survey. BMJ 328: 801–806. 15070637 10.1136/bmj.328.7443.801 PMC383376

[15] NFHS-3 (2007) National Family Health Survey (NFHS-3), 2005–06: India. International Institute for Population Sciences and Macro International Mumbai, India 1: 590.

[16] Rani M, Bonu S, Jha P, Nguyen SN, Jamjoum L (2003) Tobacco use in India: prevalence and predictors of smoking and chewing in a national cross sectional household survey. Tob Control 12: e4. 14660785 10.1136/tc.12.4.e4 PMC1747786

[17] Kolappan C, Gopi PG, Subramani R, Narayanan PR (2007) Selected biological and behavioural risk factors associated with pulmonary tuberculosis. Int J Tuberc Lung Dis 11: 999–1003. 17705978

[18] Gajalakshmi V, Peto R, Kanaka TS, Jha P (2003) Smoking and mortality from tuberculosis and other diseases in India: retrospective study of 43000 adult male deaths and 35000 controls. Lancet 362: 507–515. 12932381 10.1016/S0140-6736(03)14109-8

[19] Rooban T, Madan Kumar PD, Ranganathan K (2010) Reach of mass media among tobacco users in India: a preliminary report. Indian J Cancer 47 Suppl 153–58. 10.4103/0019-509X.63869 20622415

